# Alterations in white matter fractional anisotropy in subsyndromal perimenopausal depression

**DOI:** 10.1186/s12888-014-0367-8

**Published:** 2014-12-24

**Authors:** Xianglan Wang, Jiong Tao, Lingjiang Li, Zhiyong Zhong, Sha Liu, Tianzi Jiang, Jinbei Zhang

**Affiliations:** Mental Health Institute of the Second Xiangya Hospital, National Technology Institute of Psychiatry, Key Laboratory of Psychiatry and Mental Health of Hunan Province, Central South University, Changsha, 410011 China; Department of Psychiatry, the Third Affiliated Hospital of Sun Yat-sen University, Guangzhou, 510630 China; Shenzhen Kangning Hospital of Guangdong Province, Shenzhen, 518003 China; Department of Radiology, Guangzhou Brain Hospital, Guangzhou, 510370 China; Brainnetome Center, Institute of Automation, Chinese Academy of Sciences, Beijing, 100190 China; National Laboratory of Pattern Recognition, Institute of Automation, Chinese Academy of Sciences, Beijing, 100190 China; Queensland Brain Institute, University of Queensland, Brisbane, Queensland 4072 Australia; Key Laboratory for NeuroInformation of Ministry of Education, School of Life Science and Technology, University of Electronic Science and Technology of China, Chengdu, 610054 China

**Keywords:** White matter, Fractional anisotropy, Estrogen, Perimenopause, Depressive disorders

## Abstract

**Background:**

Subsyndromal depression (SSD) is considered as a predictor for future depressive disorders, however whether white matter abnormalities are involved in the high-susceptibility of women to depressive disorders during perimenopause is unknown. The purpose of this study was to investigate fractional anisotropy (FA) in the white matter of the whole brain in perimenopausal women with SSD using diffusion tensor imaging (DTI).

**Methods:**

In a cross-sectional study, 24 perimenopausal women with SSD and 24 other age-, education-, and body mass index-matched healthy women underwent DTI. A voxel-based analysis was used to elucidate regional FA changes at a voxel threshold of *p* < 0.001 with an extent threshold of *k* > 127 voxels (*p* < 0.05, AlphaSim correction). Subsequently, correlation analyses were performed between mean FA values in significant brain regions and plasma estradiol level.

**Results:**

Compared to healthy controls, women with SSD exhibited significantly lower FA values in the left insula, while higher FA values were observed in the left ventral lateral thalamus and left and right brainstem in the midbrain. In subjects with SSD, the mean FA value in the left insula was positively correlated to plasma estradiol levels (*r* = 0.453, *p* = 0.026) (uncorrected).

**Conclusions:**

Our findings indicate altered microstructures in white matter of the insula and subcortical regions may be associated with the high susceptibility of perimenopausal women to depressive disorders. Estrogen may modulate the white matter microstructure of the insula.

## Background

Numerous studies have confirmed that in women, perimenopause is a vulnerable window to depressive disorders [1]. The probability of a high Center for Epidemiological Studies of Depression scale (CES-D) score was four times greater during perimenopause than during the premenopausal phase [2]. One study found that perimenopausal women were twice as likely to experience significant depressive symptoms compared to premenopausal women, and major depressive disorder (MDD) occurred in 9.5% of premenopausal and 16.6% of perimenopausal women [3]. However, the causes of perimenopausal depression remain unclear. Although multiple factors may be involved, such as vasomotor symptoms, sleep disturbance, stressful life events, personal psychosocial features, and endocrine changes [1,4,5], few studies have focused on the structural or functional brain aberrancies of perimenopausal depression.

In addition to depressive disorders, such as MDD and dysthymia, which can be diagnosed by criteria such as the Diagnostic and Statistical Manual of Mental Disorders, fourth edition (DSM-IV, American Psychiatry Association, Washington, D.C., 1994), a quite common subsyndromal, subclinical, or subthreshold, but clinically undiagnosable, depressive condition exists in which people experience at least one depressive symptom (insufficient for diagnosis) or inadequate persistence (shorter than 2 weeks) and demonstrate significantly high scores on depressive symptom scales [6-8]. Moreover, subsyndromal depression (SSD) may be a remarkable predictor for future MDD, and people with SSD are highly susceptible to depressive disorders [9,10]. The Zung Self-Rating Depression Scale (ZSDS) is used globally in clinical or research assessments for depressive symptoms and depression intensity. A ZSDS index score of more than 50 has been set as the significance threshold for depression [6]. Therefore, we presumed that perimenopausal women with SSD had a higher susceptibility to perimenopausal depression than those without depressive symptoms according to ZSDS.

Distinct biological change occurring during perimenopause is the programmed aging of the hypothalamic-pituitary-gonadal axis (HPG) system. Main changes include great variations in plasma sex hormone levels, especially the fluctuation of estradiol (E2) at a higher level than in premenopause [11]; and effects on the central nervous system by the sex hormones such as estrogen and androgen [12]. Therefore, sex hormones are thought to play an important role in the pathology of perimenopausal depression. The estrogen withdrawal hypothesis for perimenopausal depression associates a decline in estrogen directly with biochemical changes in the brain and postulates that the estrogen insufficiency lead to depression [13]. However, majority of previous studies on the direct association between perimenopausal depression and plasma sex hormones have shown inconsistent results [2,14-16].

Magnetic resonance imaging (MRI) is a noninvasive detection technique that has been successfully used in investigations of brain structure and function in vivo in both healthy people and people with illnesses. DTI, an MRI technique used to study water diffusion in tissue, can provide microstructural information on nerve fiber connectivity and integrity in the white matter of the brain. Fractional anisotropy (FA) of the directionally preferential water molecule diffusivity is usually used to assess the overall white matter integrity [17]. Voxel-based analysis (VBA) is a common whole-brain analytic technique that is used to explore the local FA differences between groups, with the advantage of having less investigator bias from region-of-interest (ROI) selection [18]. Ma et al. [19] reported significantly decreased FA in the right middle frontal gyrus, left lateral occipitotemporal gyrus, and subgyral and angular gyri of the right parietal lobe in young adults with MDD. Other DTI studies also revealed brain locations of decreased FA in MDD patients, namely the white matter fascicles connecting the prefrontal cortex within cortical (frontal, temporal, and occipital lobes) and subcortical areas (amygdala and hippocampus) [20-24]. These findings were consistent with previous structural and functional MRI studies that identified brain abnormalities in patients with MDD; for example, an abnormal emotion processing and regulation system centered on the medial prefrontal-limbic network including the amygdala, hippocampus, anterior cingulate gyrus, subgenual cortex, and medial prefrontal cortex [25]. However, a study with a larger sample size (134 patients with MDD vs. 54 healthy controls) showed that patients with MDD had no significant differences in FA, radial diffusivity (RD), mean diffusivity, and axonal diffusivity compared to healthy controls [26]. Even so, a preliminary research using DTI on subjects with subclinical depression (9 men and 12 women, aged 37–71 years, CES-D score of more than 16, without a history of neurological or psychiatric illness) performed voxel-based morphometry (VBM) to calculate gray matter volume and tract-specific analyses based on VBM. This study showed decreased volumes in the bilateral anterior cingulate gyri and right rectal gyrus, and a positive correlation between the RD value of the right anterior cingulum and the CES-D score in the subclinically depressed women [27]. In another study on 810 community-dwelling adult participants by the same authors, they found a reduction in FA in the right dorsal anterior cingulate region and a positive correlation between the RD value in the same region and CES-D scores in 271 women from 24 to 81 years old [28].

The present study investigated the mechanisms underlying the high susceptibility of certain perimenopausal women to depressive disorders. We used diffusion tensor imaging of the brain in combination with correlation analyses between abnormal brain regions and plasma E2 level. We hypothesized that the increase vulnerability to depression of certain perimenopausal women would be related to aberrant white matter microstructure, and these alterations would be associated with plasma E2 levels. Thus, we compared the FA values in the white matter of the whole brain between perimenopausal women with SSD and healthy controls, and performed correlation analyses between the mean FA values of aberrant brain regions and plasma E2 in women with SSD. The results of this study provide important clues for etiological and neuroimaging research on depressive disorders in the future.

## Methods

### Subjects and depressive symptom assessment

The study was approved by the Ethics Committee of the Second Xiangya Hospital of Central South University. All subjects were recruited by placing advertisements in the Third Affiliated Hospital of Sun Yat-sen University and in some nearby communities from October 2012 to July 2013. Forty-eight volunteers participated in this study, and they all had signed informed consent forms (ICF) before the study. The inclusion criteria were as follows: (1) women from 45 to 55 years old; (2) meeting the criteria of perimenopause according to the Stages of Reproductive Aging Workshop system [29]; (3) with an education level of more than 5 years in elementary school in case of failure to complete the self-report scale and ICF; and (4) Han Chinese and right-handed. Exclusion criteria included: (1) a history of medical events that may significantly affect the study outcome, such as metabolic or endocrine disease; (2) a personal or family history of mental disorders, or presence of metal implants or metal objects in the body that cannot be removed (e.g., implanted ceramic dentals); and (3) recent notable negative life events, such as divorce, loss of job, fortune and/or important relatives.

Firstly, we used the ZSDS, a 20-item self-report questionnaire, to assess depression symptoms and the depressive severity in all participants [30]. A cut-off index score of 50 was used to determine if there were the depression symptoms [6]. Secondly, we used the Chinese version of the Mini-International Neuropsychiatric Interview (MINI) [31,32] for clinical assessment of depressive disorders in participants with ZSDS index score more than or equal to 50. Twenty-four participants with ZSDS scores more than or equal to 50 and not met the criteria for MDD according to MINI were assigned in the SSD group. Twenty-four other age-, education-, and body mass index (BMI)-matched participants with ZSDS score less than 50 were assigned in the group of healthy controls (HC).

### Sex hormone examination

Every subject meeting the inclusion criteria had an appointment scheduled specifically according to her menstrual cycle: one of the first 6 days of menstruation for women with identifiable menstrual cycles, and any day at their convenience for women with irregular menstrual cycles, i.e. the period interval more than 2 months. During their appointments, subjects were tested for plasma E2 levels. The plasma concentrations of E2 were measured using the Siemens ADVIA Centaur XP Immunoassay System (ADVIA Centaur XP, Siemens Healthcare Diagnostics Inc., New York, USA) in the Department of Clinical Laboratories in the Third Affiliated Hospital of Sun Yat-sen University.

### MRI Data acquisition and processing

Images were acquired using a 3.0-Tesla Philips Achieva MR scanner (PHILIPS Achieva X-series, Philips Medical, Best, Netherlands). A single-shot, spin echo-echo planar imaging sequence (repetition time = 8,500 ms, echo time = 70 ms, slice thickness = 2.0 mm, no gap, 75 coronal slices, voxel size = 2 × 2 × 2 mm^3^, field of view = 256 mm × 256 mm, scan matrix = 128 × 128, flip angle = 90°) was used to acquire the DTI images. Diffusion-sensitizing gradients were applied along 33 non-collinear directions (b = 1000 s/mm^2^), and one non-diffusion weighted image (b = 0) was acquired. Subjects were given soft earplugs, positioned comfortably in the coil, and instructed to relax and remain still. Head motion was minimized with foam pads. High-resolution three-dimensional (3D) T1-weighted images were acquired using a fast field echo-3D T1 sequence (repetition time = 8 ms, echo time = 4 ms, 188 sagittal slices, slice thickness = 1.0 mm, no gap, flip angle = 7°, voxel size = 1 × 1 × 1 mm^3^).

Diffusion tensor images were pre-processed using previously published methods [33]. The diffusion dataset was pre-aligned to correct for head motion and the effects of gradient coil eddy currents using software tools from the FMRIB software library (FSL; http://www.fmrib.ox.ac.uk/fsl). After these steps, the diffusion tensor at each voxel was calculated using the FMRIB diffusion toolbox in FSL. The resulting FA images were transformed into Montreal Neurological Institute (MNI) standard space with Statistical Parametric Mapping (SPM8) software (Wellcome Department of Cognitive Neurology, London, UK) by means of the following steps: the b = 0 images were coregistered with the 3D T1 image of the individual, the same coregistration parameters were applied to the FA maps (in the same space as the b = 0 images). Each individual’s T1 image was then normalized to the SPM T1 template (in MNI standard space), and the same normalization parameters were then applied to the coregistered FA images using the "Preserve Concentration" option. Subsequently, FA images were smoothed with an 8 mm full-width at half-maximum Gaussian kernel in order to decrease spatial noise and compensate for the inexact nature of normalization. Finally, all images were re-sampled with a voxel size of 2 × 2 × 2 mm^3^. For using in the two-sample t test, a binary white matter mask was made by calculating a mean FA image with a threshold FA value of 0.25 from FA images of all participants.

### Statistical analysis

#### FA differences

VBA was performed using SPM8 software. FA maps were compared between the SSD and HC groups using two-sample *t-*tests. The white matter mask made in processing was used to restrict the search volume for analysis. The statistical threshold for each voxel was set at *p* < 0.001 with an extent threshold of *k* > 127 voxels (1016 mm^3^), which yield a corrected threshold of *p* < 0.05, determined by the Resting-State fMRI Data Analysis Toolkit (REST) (http://restfmri.net/forum) AlphaSim program (parameters were: FWHM = 9.9 mm, 10.6 mm, 9.9 mm, rmm = 5, number of Monte Carlo simulations = 5000) [34]. To quantify the changes in the affected regions, we constructed the ROIs mask by setting the peak coordinate of each significant cluster in the group comparison as the center of a sphere with the radius = 6 mm, respectively. For further correlation analyses, the mean FA value of each ROI was extracted from the FA maps of every subject in the SSD group using the utility for ROI time course extraction in the REST [34].

#### Correlation analyses

The Shapiro-Wilk test showed that data of the plasma E2 (*W* = 0.927, *p* = 0.083), FA values in the left insula (*W* = 0.965, *p* = 0.543), midbrain (*W* = 0.980, *p* = 0.898), and left thalamus (*W* = 0.962, *p* = 0.480) were probably normally distributed. Thus, the Pearson correlation was used in the correlation analyses between E2 and FA values of significant regions within SSD subjects. The two-sample *t-*test was performed to compare the age, education level, BMI, ZSDS scores, and plasma concentration of E2 between the SSD and HC groups, respectively. All statistics in this section were carried out using the Statistical Package for the Social Sciences (SPSS 16.0; SPSS Inc., Chicago, IL) with a significance level of *p* < 0.05 (uncorrected).

## Results

### Subject characteristics

The demographic characteristics and clinical data of the two groups are shown in Table 1. The SSD group had a mean ZSDS score of 55.33 (standard deviation [SD], 4.72) and included 16 mild and 8 moderate to severe depressive cases. The HC group had a mean ZSDS score of 37.38 (SD, 5.19; *t* = 12.546, *p* < 0.0001). There were no significant differences in age, education level, BMI, or E2 between the two groups (*p* > 0.05).

**Table 1:** **Demographic, clinical assessment and sex hormone data of the participants, as well as group comparisons**

	**SSD (N = 24)**	**HC (N = 24)**	**Statistics**	***P*** **value**
Age (years, mean ± SD)	47.88 ± 2.17	47.29 ± 2.03	*t* = 0.961	0.342
Education (years, mean ± SD)	6.98 ± 1.60	7.56 ± 1.31	*t* = 1.378	0.175
BMI (kg/m^2^, mean ± SD)	24.78 ± 2.92	23.63 ± 2.52	*t* = 1.461	0.151
ZSDS index score (mean ± SD)	55.33 ± 4.72	37.38 ± 5.19	*t* = 12.546	<0.0001
E2 (pmol/L, mean ± SD)	328.61 ± 217.20	358.60 ± 249.71	*t* = 0.444	0.659

SSD: subsyndromal depression; HC: healthy control; BMI: body mass index; ZSDS: Zung Self-rated Depression Scale; E2: estradiol.

### FA values differ between SSD and HC subjects

**Figure 1:**
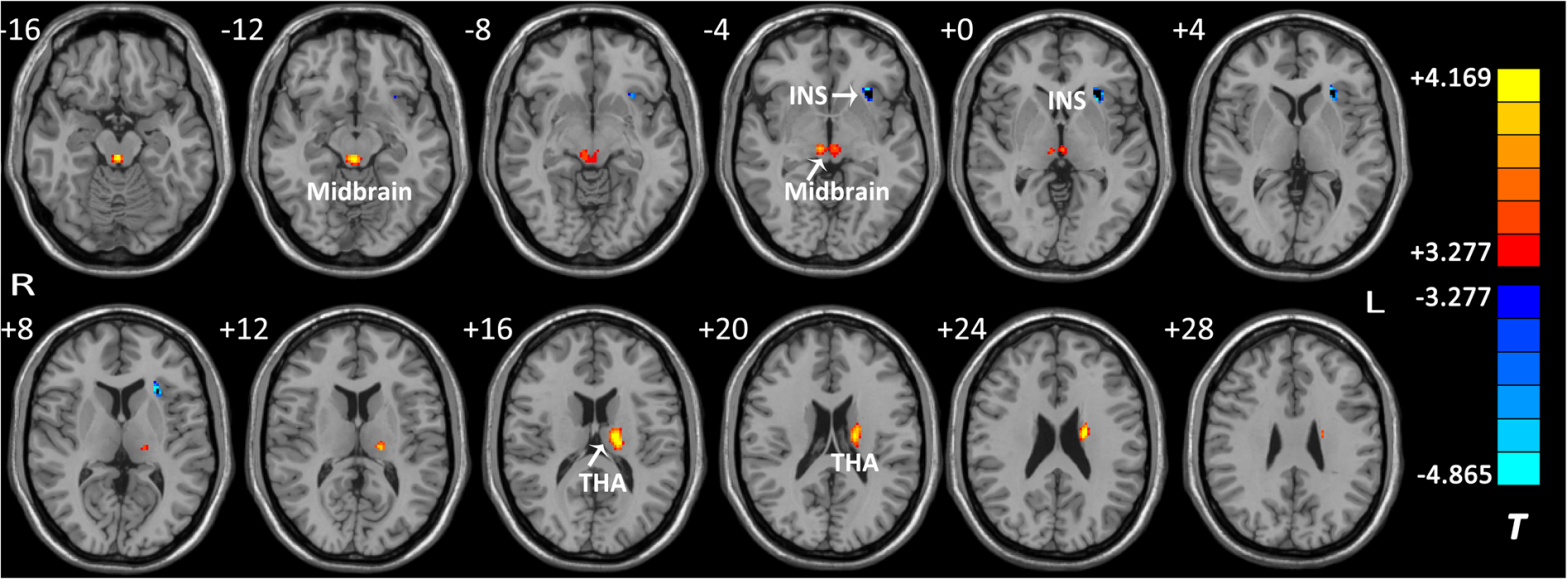
**Brain regions with altered fractional anisotropy (FA) in subjects with subsyndromal depression compared to healthy controls.** A significant height threshold of *p* < 0.001 with an extent threshold of *k* > 127 voxels (1016 mm^3^) (*p* < 0.05, corrected by AlphaSim correction) was set for the two-sample *t-*test*.* Warm colors represent increased FA values (SSD > HC), and cool colors represent decreased FA values (SSD < HC). INS: left insula; THA: left thalamus; Midbrain: left and right brainstem in midbrain. The details of altered regions are listed in Table 2.

Brain regions with altered FA in white matter are shown in Figure 1 and Table 2. Compared to the HCs, perimenopausal women with SSD had decreased FA values in the left anterior insula (Brodmann area [BA] 13) [35]. Meanwhile, women with SSD showed increased FA values in the left ventral lateral thalamus extending to the extra-nuclear area, and left and right brainstem in the midbrain.

**Table 2:** **The FA values of different brain regions between the SSD and HC groups**

**Different regions**	**Hemisphere**	**BA**	**Cluster size**	**Peak coordinate (x, y, z)**	***T*** **-value** ^**a**^
SSD < HC					
INS	L	13	132	−30, 22, −2	4.865
SSD > HC					
Midbrain	L/R		221	2, −30, −14	4.169
THA	L		226	−18, −18, 16	4.124

^a^Significance threshold: *df* = 46, *p* < 0.001 (*T* = 3.277) with *k* >127 voxels (*p* < 0.05, corrected by AlphaSim correction). FA: fractional anisotropy; SSD: subsyndromal depression; HC: healthy control; L: left; R: right; BA: Brodmann area; INS: insula; THA: thalamus.

### Correlation analyses between the mean FA values of altered brain regions and plasma E2 level within SSD subjects

**Figure 2:**
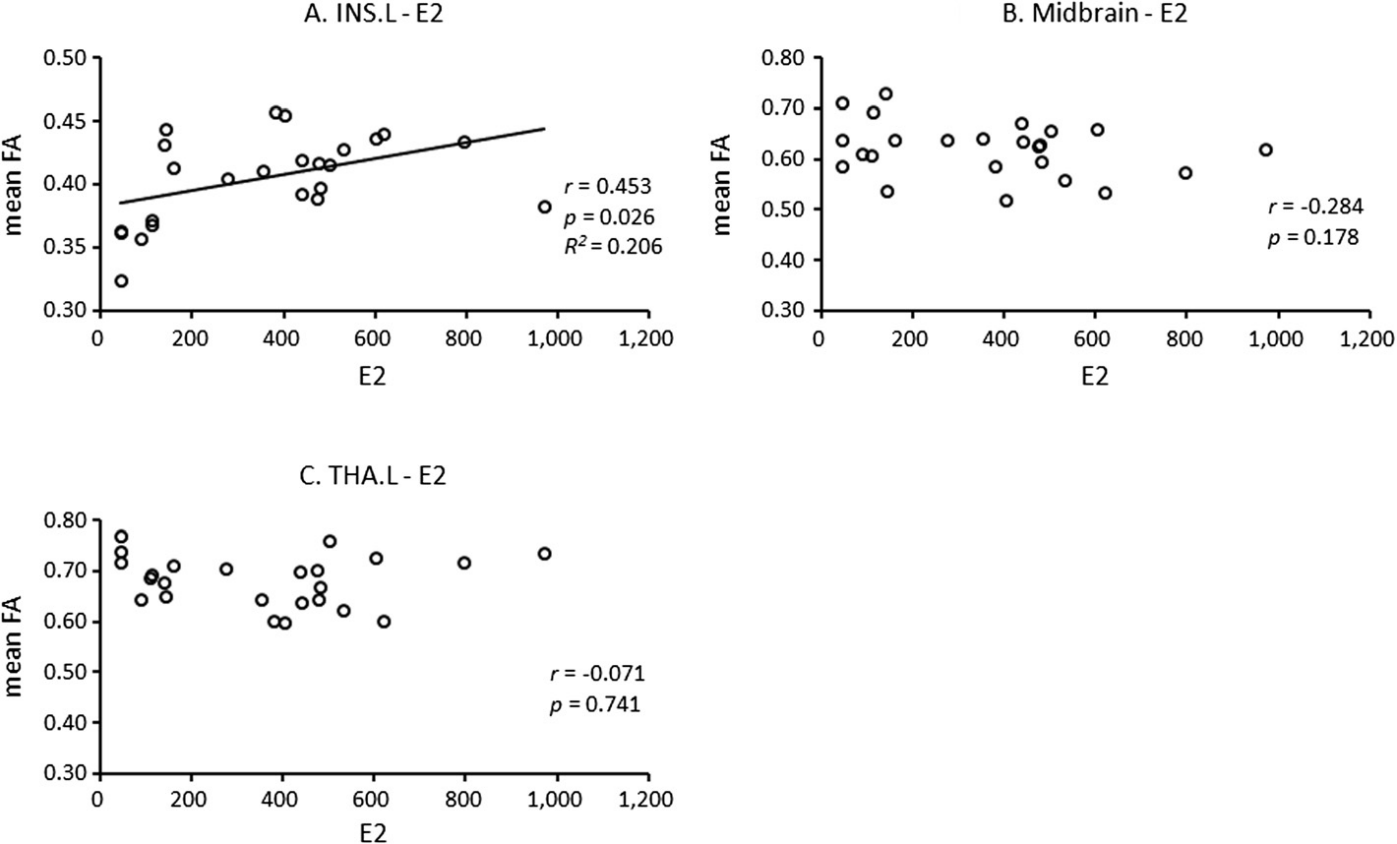
**Scatterplots between mean FA values in altered brain regions and plasma E2 level within SSD subjects. (A)** Correlation between mean FA values in INS.L and plasma E2; **(B)** Correlation between mean FA values in Midbrain and plasma E2; **(C)** Correlation between mean FA in THA.L and plasma E2. Abbreviations: FA: fractional anisotropy; SSD: subsyndromal depression; E2: estradiol; INS.L: left insula; Midbrain: left and right brainstem of midbrain; THA.L: left thalamus.

Pearson bivariate correlation analyses showed a positive relationship between the mean FA value of the left insula and plasma E2 levels (*r* = 0.453, *p* = 0.026) (Figure 2). There were no significant correlations between plasma E2 and mean FA values in the midbrain (*r* = −0.284, *p* = 0.178) or left thalamus (*r* = −0.071, *p* = 0.741)(Figure 2).

## Discussion

In this study, we focused on women during the perimenopause, which has been considered as a vulnerable window to depressive disorders. Women with SSD were thought more susceptible to depressive disorders than those without depressive symptoms. To investigate regional abnormalities of the white matter in perimenopausal women with SSD, DTI was used and FA was calculated and statistically analyzed. We found decreased FA values in the insula and increased FA values in the thalamus and midbrain. Meanwhile, FA values in the insula were in concordance with plasma estradiol level in subjects with SSD. These findings suggest that alterations in the white matter of emotion-related brain regions and subcortical regions may be involved in the high-susceptibility of perimenopausal women to depressive disorders.

Reduced FA has been considered to be a clue to impaired myelin fiber integrity in the white matter [17]. In the present study, we found decreased FA in the left anterior insula (Figure 1 and Table 2). The insula, especially the anterior insula, has been considered as an integral hub for mediating dynamic interactions between other large-scale brain networks, such as the default mode network, salient network, and central executive network. Therefore, the insula is thought to be involved in emotional processing, cognitive control, self-awareness, and homeostasis, based on its structural and functional connectivity with the limbic system, prefrontal lobe, and subcortical structures (e.g., the thalamus and the midbrain) [36]. In patients with MDD, reduced gray matter volume has been noted in the insula [37-39], but few studies have reported abnormal FA values in the white matter of this region. Our finding indicates that the microstructure of white matter in the insula maybe was abnormal in perimenopausal women with even depressive symptoms. Whether structural or functional abnormalities in the insula are the early pathological changes of perimenopausal depression, still needs to be validated in future studies.

Moreover, the present study showed a positive relationship between mean FA value in the left insula and plasma E2 levels (*r* = 0.453, *p* = 0.026) (uncorrected) (Figure 2). Estrogen has widespread effects on the human brain, especially the emotional circuitry [40], and may have neuroprotective and antidepressant roles in MDD [41,42]. Additionally, Berent-Spillson et al. reported that long-term exposure to exogenous estrogen maybe affect the functional activity of the insula. They found that menopausal women using conjugated equine estrogens for more than ten continuous years had increased activation in the frontal and parietal cortices, insula, hippocampus, and cingulate during the visual work memory task compared to never-users [43]. Until recently, few studies have focused on the association between alterations in the structure or function of brain regions and endogenous E2 level in patients with depressive disorders. Our finding suggest that circulating E2 level may act on the microstructure of the white matter in emotion-related brain regions, and to an extent, support the estrogen withdrawal hypothesis for perimenopausal depression [13].

Unlike previous studies on DTI in patients with depressive disorders, we also discovered two subcortical regions with higher FA values in subjects with SSD compared to healthy controls, including the left and right brainstem in the midbrain and the left thalamus (Figure 1 and Table 2). In patients with euthymic bipolar disorder and attention deficit/hyperactivity disorder (ADHD), elevated FA in white matter has been commonly reported [44,45], and Silk et al. [46] suggested that the greater FA values observed in patients with ADHD may represent an abnormal reduction in the degree of neuronal branching within significant areas. Human midbrain contains several important nerve tracts involved in up or down transmissions of sensory or motor information, reticular formation and critical nuclei producing monoamine neurotransmitters such as the dopamine, norepinephrine, and serotonin [47]. The monoamine-deficiency hypothesis presumes that norepinephrine and serotonin have critical roles in the mechanisms of the pathogeny and treatment of depression [48]. Meanwhile, the thalamus is generally believed to act as a relay between different subcortical areas and the cerebral cortex. Previous studies have found increased thalamic functional connectivity in the default-mode network of depressed subjects [49], and increased thalamic metabolism during depression episodes [50,51]. Collectively, our findings of increased FA values suggest that aberrant white matter microstructures in the midbrain and thalamus in SSD subjects may be involved in perimenopausal depression.

The sample size of this study was relatively small to determine the alteration in the white matter of brain and the relationship between plasma sex hormone levels and significant brain regions. This study focused on perimenopausal women with SSD and healthy controls; however, we did not include perimenopausal women with MDD or premenopausal women with depressive disorders for comparison. Future research using a larger sample size and a more comprehensive range of participants is needed to validate our findings. In addition, other methods for the microstructural analysis in the brain white matter, such as TBSS, maybe offer more informative, precise, and inspiring details about the brain mechanism of the subsyndromal perimenopausal depression.

## Conclusions

In conclusion, the results of the present study indicate that the altered microstructures in white matter of the insula and subcortical regions may be associated with the high-susceptibility of perimenopausal women to depressive disorders. Meanwhile, estrogen may modulate the white matter microstructure of the insula.
